# Genomic identification of recipient-derived hepatocytes in liver transplantation: a hypothesis linking graft repopulation to immune tolerance

**DOI:** 10.3389/fimmu.2025.1642451

**Published:** 2025-07-31

**Authors:** Seoung Hoon Kim

**Affiliations:** Organ Transplantation Center, National Cancer Center, Goyang-si, Gyeonggi-do, Republic of Korea

**Keywords:** liver transplantation, recipient-derived hepatocytes, graft repopulation, operational tolerance, genomic chimerism, immunosuppression minimization

## Abstract

**Background:**

Operational tolerance, defined as stable liver graft function without immunosuppression, has been observed in select transplant recipients. While immune regulatory mechanisms have been implicated, the biological processes underlying tolerance remain incompletely understood. Notably, recipient-derived hepatocytes have been shown to progressively repopulate donor livers, raising the possibility that this histological change may contribute to tolerance induction.

**Hypothesis:**

This hypothesis suggests that progressive replacement of donor hepatocytes by recipient-derived cells reduces donor alloantigen exposure, thereby attenuating allo-immune responses and enabling stable graft acceptance without pharmacologic immunosuppression. This phenomenon could be detected through Y-chromosome–specific assays in sex-mismatched transplants or via donor-recipient genomic profiling in all cases.

**Supporting evidence:**

The liver’s intrinsic regenerative capacity permits continuous hepatocyte turnover and engraftment of recipient-derived cells, particularly under conditions of chronic low-grade injury. Clinical reports have documented the presence of recipient-derived hepatocytes in liver allografts, and operational tolerance has been associated with decreased donor-derived cell-free DNA and reduced allo-immune activation. Although techniques such as FISH and qPCR targeting the Y-chromosome are effective in sex-mismatched cases, broader applicability requires STR or SNP-based genotyping. Integrating these genetic approaches with hepatocyte-specific methylation or transcriptomic profiling may significantly improve the accuracy and clinical relevance of recipient-derived hepatocyte detection.

**Implications:**

This hypothesis, if validated, could shift the conceptual model of transplant tolerance from solely immune regulation to a dynamic process of histological replacement. It may also lead to biomarker-driven strategies for immunosuppression withdrawal support novel diagnostic approaches to confirm operational tolerance in appropriate candidates.

## Introduction

Operational tolerance refers to the long-term survival of a transplanted organ without ongoing immunosuppression, accompanied by stable graft function and the absence of rejection (1). While rare in most solid organ transplants, liver grafts uniquely demonstrate a relatively high propensity for spontaneous tolerance, with reported rates ranging from 20% to 40% in carefully selected immunosuppression withdrawal trials (2–5).

To date, proposed mechanisms for operational tolerance have centered on immunological regulation, including expansion of regulatory T cells, altered dendritic cell phenotypes, and upregulation of inhibitory immune checkpoints. However, these explanations focus solely on immune cell behavior and do not address the potential biological alterations occurring within the graft itself.

We hypothesize that progressive replacement of donor-derived hepatocytes by recipient-derived hepatocytes within the graft contributes significantly to tolerance by decreasing the burden of donor alloantigen presentation. This process, largely overlooked in prior tolerance studies, may constitute a fundamental biological mechanism supporting stable graft acceptance.

## Recipient-derived hepatocyte repopulation: mechanisms and evidence

Hepatocyte turnover occurs naturally in healthy livers and is accelerated following injury or inflammation. In the setting of liver transplantation, continuous low-grade immune-mediated injury, cellular senescence, and regenerative demands create a permissive environment for recipient-derived cells to engraft and expand within the donor liver. Several studies have shown that recipient-derived hepatocytes can repopulate donor livers over time, suggesting a natural mechanism of graft adaptation (6–9).

Evidence from animal models and clinical case series has demonstrated that bone marrow-derived or endogenous recipient hepatocytes can populate liver allografts over time (10–12). Translating these findings to human transplantation has been limited by the challenge of identifying recipient-derived cells within donor tissue.

Fluorescence *in situ* hybridization (FISH) for sex-mismatched transplants, and short tandem repeat (STR) profiling or single nucleotide polymorphism (SNP) genotyping in all donor–recipient combinations provide a unique opportunity to confirm the presence of recipient-derived hepatocytes in liver grafts.

However, their potential role in operational tolerance has yet to be investigated.

## Hypothesis: repopulation drives tolerance

We propose that as recipient-derived hepatocytes progressively replace donor hepatocytes, the overall alloantigenic burden within the graft diminishes. This chimerism could serve as both a biomarker and a mechanistic contributor to immune adaptation and tolerance (13, 14). The timeframe over which this repopulation occurs, however, remains poorly defined, and may vary significantly between individuals depending on factors such as immune activity, graft injury, and regenerative demand. While the phenomenon of recipient-derived hepatocyte repopulation has been previously observed, this study aims to address a critical translational gap in the application of hepatocyte repopulation as a biomarker for immunosuppression management. No method or biomarker is currently established in clinical practice to detect such repopulation and guide immunosuppression. The proposed hypothesis introduces a practical and generalizable approach using genomic profiling to detect recipient-derived hepatocytes, including in sex-matched liver transplantation where Y-chromosome–based methods are not applicable.

**Figure 1:**
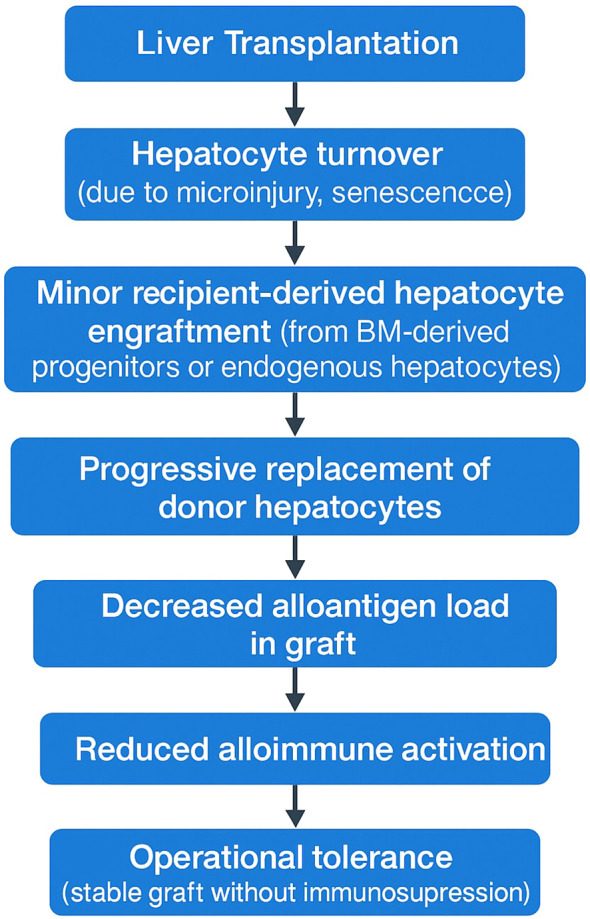
Proposed mechanistic model linking recipient-derived hepatocyte repopulation to operational liver transplant tolerance.

The proposed sequence begins with liver transplantation and subsequent immune-mediated graft injury, followed by hepatocyte turnover and minor engraftment of recipient-derived cells. Over time, this leads to gradual replacement of donor hepatocytes by recipient-derived hepatocytes, resulting in a progressive reduction of donor alloantigen load, attenuation of allo-immune surveillance and activation, and ultimately the establishment of operational tolerance (**Figure 1**).

This conceptual framework suggests that operational tolerance is not solely an immune regulatory phenomenon but also a histological remodeling process.

## Potential experimental approaches

To investigate this hypothesis, several complementary strategies can be employed. For patient selection, recipients of either sex-matched or sex-mismatched donor livers, ideally in the context of living donor liver transplantation where long-term follow-up and protocol biopsies are feasible. In liver transplant recipients who have maintained stable graft function for at least one year, it is common clinical practice to taper immunosuppression to a minimal maintenance dose. At this stage, performing a protocol liver biopsy combined with genomic identification techniques — such as FISH, STR, or SNP-based assays — could identify the degree of recipient-derived hepatocyte repopulation within the graft. If substantial repopulation is confirmed, it may justify complete immunosuppression withdrawal in select patients, supporting a precision, biomarker-guided immunosuppression management strategy.

Detection techniques include fluorescence *in-situ* hybridization (FISH) for Y-chromosome detection in sex-mismatched transplants, quantitative PCR (qPCR) targeting Y-specific sequences such as SRY and DYZ1, and short tandem repeat (STR) or SNP-based genomic analysis to distinguish donor and recipient DNA profiles for cell origin determination. Additionally, dual immunostaining with hepatocyte-specific markers like Hepatocyte Nuclear Factor 4 alpha (HNF4α) can be employed to confirm the hepatocellular identity of repopulating cells.

**Figure 2:**
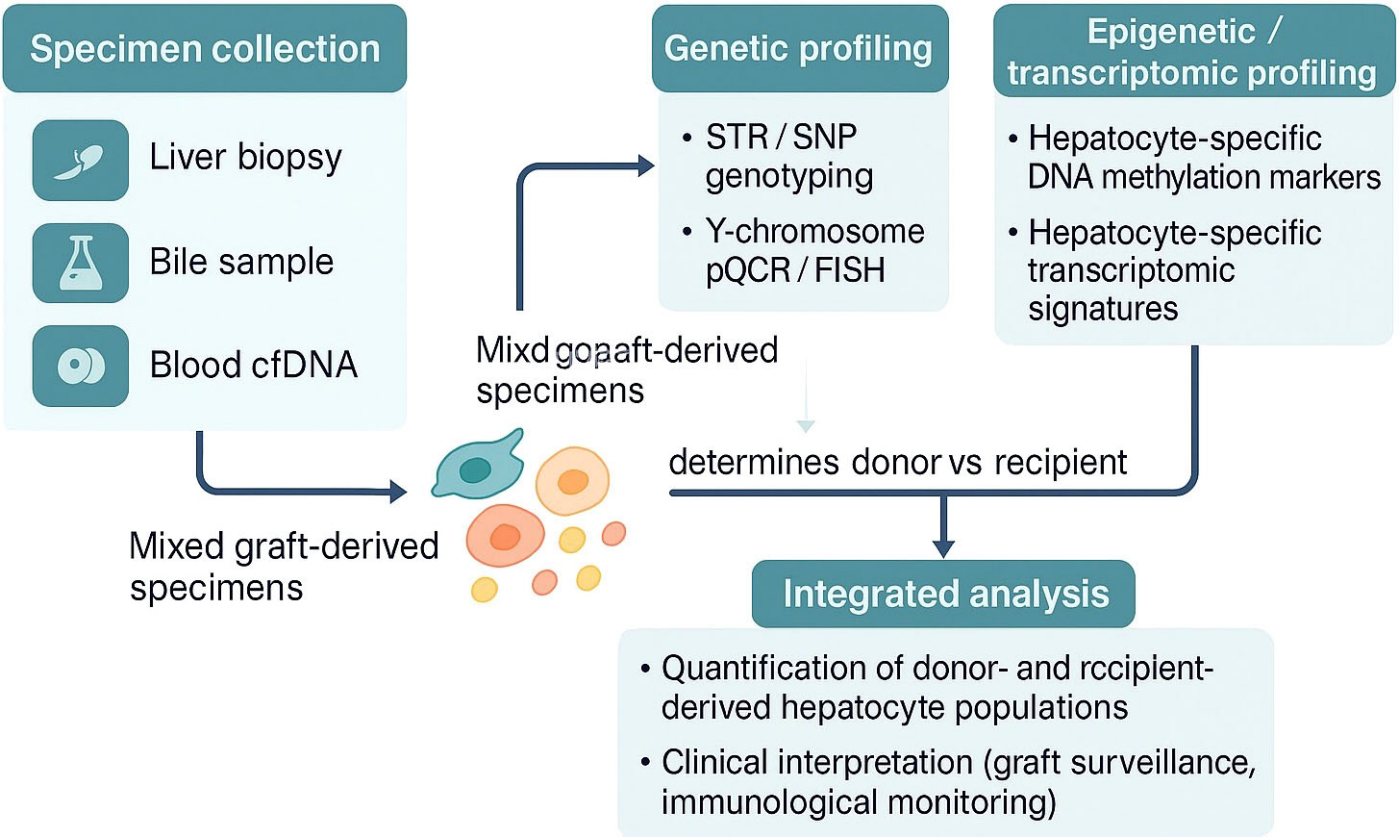
Proposed integrative workflow for determining donor- or recipient-derived hepatocyte origin in liver transplant recipients.

A conceptual workflow is proposed for identifying the cellular origin of hepatocytes in liver transplant recipients (**Figure 2**). Mixed post-transplant specimens (e.g., liver biopsy, bile, blood-derived cell-free DNA) contain donor- and recipient-derived hepatocytes along with other non-parenchymal cells. Genetic assays such as STR, SNP genotyping, or Y-chromosome-specific qPCR/FISH enable determination of donor versus recipient origin but cannot differentiate between hepatocytes and other cell types. By integrating hepatocyte-specific methylation markers or transcriptomic profiles, the specificity of hepatocyte origin detection is improved. This approach facilitates the sensitive detection of minor cell populations, such as recipient-derived hepatocytes, and offers a framework for clinical biomarker development and mechanistic investigations into graft repopulation and immune tolerance.

## Discussion

The identification of donor- and recipient-derived hepatocytes following liver transplantation holds significant clinical value for monitoring graft cell turnover, assessing graft repopulation, and understanding immune-mediated processes. While this concept has been widely explored in experimental models and sporadically applied in clinical studies, its practical application in routine transplant medicine remains constrained by technical complexity, lack of validated assays, and limited integration into clinical workflows.

The proposed biological sequence involves initial immune-mediated graft injury following transplantation, hepatocyte turnover, and minor recipient-derived cell engraftment, followed by gradual replacement of donor hepatocytes with recipient-derived cells. This would progressively reduce the donor alloantigen burden, diminish allo-immune surveillance and activation, and ultimately lead to the establishment of operational tolerance. Since the kinetics of recipient-derived hepatocyte repopulation remain poorly characterized, longitudinal analyses incorporating protocol liver biopsies and serial monitoring of donor-derived cell-free DNA would be helpful to define the timing, rate, and extent of this process in transplant recipients. Moreover, a critical unmet need is the establishment of an evidence-based consensus regarding the threshold level of recipient-derived hepatocyte repopulation necessary to safely define operational tolerance and justify immunosuppression withdrawal. Future prospective studies should focus on determining these clinically actionable cut-off values by correlating repopulation rates with graft outcomes, immune profiles, and tolerance-associated biomarkers.

Genetic profiling techniques such as short tandem repeat (STR) analysis, single nucleotide polymorphism (SNP) genotyping, and Y-chromosome-specific fluorescence *in situ* hybridization (FISH) or quantitative polymerase chain reaction (qPCR) have long been established in forensic science for individual identification. These methods provide highly accurate and reproducible results when applied to pure DNA samples obtained from distinct individuals or tissues. In the context of liver transplantation, however, several unique challenges complicate their application.

First, post-transplant specimens—whether obtained from liver biopsy, bile, or blood-derived cell-free DNA—are inherently composed of mixed cell populations. These samples contain varying proportions of donor- and recipient-derived hepatocytes, cholangiocytes, immune cells, and other stromal components, making the unambiguous attribution of genetic material to specific cell types difficult. Unlike forensic cases where a DNA sample typically originates from a single individual, transplant specimens require both cellular origin identification and cell type specificity, which conventional genetic assays cannot provide.

Second, while DNA-based analyses confirm the presence or absence of donor- or recipient-derived sequences, they cannot distinguish between hepatocytes and non-parenchymal cells. This limitation is particularly relevant in liver transplantation, where hepatocyte-specific changes are of primary clinical interest. To address this, cell-type-specific epigenetic signatures, such as hepatocyte-specific DNA methylation patterns or transcriptomic profiles, may be integrated with genetic profiling to improve both sensitivity and specificity. Importantly, hepatocyte-specific methylation markers do not merely improve signal specificity—they also reflect underlying biological processes such as cell differentiation, senescence, or injury, thus potentially offering both diagnostic and mechanistic insights (15, 16). Such integrative approaches have demonstrated feasibility not only in oncology and regenerative medicine (17–19), but also in solid organ transplantation (20, 21). Additionally, non-invasive approaches such as serial cell-free DNA (cfDNA) analysis from hepatic venous effluent or peripheral blood, augmented by hepatocyte-specific methylation signatures, could serve as valuable tools for real-time graft monitoring.

Third, the detection and quantification of minor cell populations, such as recipient-derived hepatocytes within a predominantly donor graft, necessitate highly sensitive assays capable of identifying low-frequency variants. Although next-generation sequencing (NGS) and digital PCR technologies offer improved sensitivity, their application for routine clinical monitoring requires standardization, validation, and cost-effectiveness studies.

Finally, while the proposed integrative strategy of combining genetic, epigenetic, and transcriptomic profiling holds conceptual promise, several translational barriers must be overcome. These include technical standardization across laboratories, definition of clinically relevant cut-off values, and prospective validation in large, well-characterized transplant cohorts. Importantly, ethical considerations surrounding genetic testing in transplantation contexts should also be carefully addressed.

Despite current technical and logistical limitations, the conceptual integration of these advanced profiling strategies offers a biologically grounded, clinically meaningful framework for improving personalized transplant care. If successfully validated, this approach may support individualized immunosuppression strategies, optimize graft surveillance protocols, and provide novel insights into the mechanisms of immune tolerance in liver transplantation.

In conclusion, recipient-derived hepatocyte repopulation represents a promising biomarker candidate for identifying transplant recipients who may achieve operational tolerance. While current genomic and epigenomic techniques show potential for detecting this phenomenon, their routine clinical use requires further validation. Defining clinically meaningful thresholds for hepatocyte repopulation and correlating them with immune tolerance and graft outcomes should be the focus of future prospective studies. This approach may ultimately support biomarker-guided immunosuppression strategies in liver transplantation.

## Data Availability

The original contributions presented in the study are included in the article/supplementary material. Further inquiries can be directed to the corresponding author.
